# Leveraging known Pacific colonisation times to test models for the ancestry of Southeast Asians

**DOI:** 10.1038/s41598-025-20856-3

**Published:** 2025-10-23

**Authors:** Mafalda Almeida, Francesca Gandini, Teresa Rito, M. George Foody, Andreia Brandão, Marisa Oliveira, Anna Olivieri, Alessandro Fichera, Gonzalo Oteo-Garcia, Zafarina Zainuddin, Ken Khong Eng, William Pomat, Jarosław Bryk, Luísa Pereira, Helen Farr, Maria Pala, Stephen J. Oppenheimer, Martin B. Richards, Pedro Soares

**Affiliations:** 1 CBMA (Centre of Molecular and Environmental Biology), Department of Biology, University of Minho, Campus de Gualtar, Braga, 4710-057 Portugal; 2 Institute of Science and Innovation for Bio-Sustainability, University of Minho, Campus de Gualtar, Braga, 4710-057 Portugal; 3 School of Applied Sciences, University of Huddersfield, Huddersfield, UK; 4 i3S — Instituto de Investigação e Inovação em Saúde, Universidade do Porto, Porto, Portugal; 5 Instituto de Patologia e Imunologia Molecular da Universidade do Porto (IPATIMUP), Universidade do Porto, Porto, Portugal; 6 Cancer Genetics Group, IPO Porto Research Center (CI-IPOP)/RISE@CI-IPOP (Health Research Network), Portuguese Oncology Institute of Porto (IPO Porto)/Porto Comprehensive Cancer Center, Porto, Portugal; 7 Dipartimento di Biologia e Biotecnologie, Università di Pavia, Pavia, Italy; 8 Analytical Biochemistry Research Centre (ABrC) and Human Identification/DNA Unit, Universiti Sains Malaysia, Penang, 11800 USM Malaysia; 9 BPP University, London, UK; 10 Papua New Guinea Institute of Medical Research, Post Office Box 60, Goroka, Papua New Guinea Papua New Guinea; 11 Centre for Maritime Archaeology, Archaeology, Southampton University, Southampton, UK; 12 School of Anthropology and Museum Ethnography, University of Oxford, Oxford, UK

**Keywords:** Pacific colonization, Lapita, Mitochondrial DNA, Polynesian motif, Molecular clock, Genetics, Evolutionary biology, Haplotypes, Population genetics, Anthropology, Archaeology, Evolutionary genetics, Population genetics

## Abstract

The most widely accepted model for the colonization of Remote Oceania by Austronesian-speaking bearers of the Lapita complex ~ 3 ka (3000 years ago) links it to a broader Neolithic expansion from China, *via* Taiwan, ~ 4.5–6 ka. However, analyses of mitochondrial DNA haplogroup B4a1a1a, prevalent among Remote Oceanians today, have both supported and challenged this scenario. Here, we analyze 1364 B4a1a1 mitogenomes (234 novel) from 68 islands and compare age estimates with radiocarbon dates for colonization. We estimate the settlement of Remote Oceania ~ 3.2 [2.7; 3.75] ka, matching radiocarbon ages, and then extrapolate the age in Near Oceania. B4a1a1a arose around the northern coasts of New Guinea at least 6 ka, following Early Holocene dispersals from Asia. Technological advances (e.g., in sailing), fueled by interaction networks alongside the arrival of Late Holocene migrants from Taiwan or ISEA and putative environmental changes, likely triggered the expansion of Lapita colonists carrying B4a1a1a from New Guinea into Remote Oceania.

**Supplementary Information:**

The online version contains supplementary material available at 10.1038/s41598-025-20856-3.

## Introduction

 The ancestry of Pacific islanders has been one of the most active and contested areas of research in archaeogenetics for three decades. Archaeological research suggests an initial arrival of the earliest ancestral modern human population in New Guinea and Australia at least 50,000 years ago (50 ka)^1^ and possibly as early as 64 ka^2–4^. This initial Early Pleistocene expansion resulted in the colonization of Near Oceania, as far as the Solomon Islands^5,6^. Large inter-island distances, in the absence of appropriate sailing technology, possibly acted as barriers to migrations further east into Remote Oceania until after ~ 3.5 ka.

According to the prevailing model, the first humans to reach Remote Oceania were associated with the so-called “out-of-Taiwan” dispersal: a large-scale expansion of rice farmers that began ~ 5–6 ka in South China and thence into Taiwan ~ 5 ka^7,8^. From there, this model suggests, Austronesian-speaking agricultural groups migrated into the Philippines ~ 4.2 ka and dispersed through the remaining Island Southeast Asia (ISEA: the Philippines, Indonesia excluding Papua, and East Malaysia) ~ 4 ka and further into Near Oceania^9–12^. This “farming/language dispersal” model^13^ has been both developed and challenged from within archaeology. For example, Green proposed the influential “Triple-I” model, positing immigration, integration and innovation, retaining the large-scale demic movements but with an important role for the local communities^14^. Spriggs, on the other hand, has proposed a version based on a kind of elite dominance rather than large-scale farming/language dispersal, in a process akin to modern globalisation, in which new identities were forged alongside the introduction of the Austronesian language across ISEA and the western Pacific^15,16^.

Most researchers consider Austronesian-speaking groups from ISEA to have been responsible (whether in whole or in part) for the emergence of Lapita^17^, a cultural complex (or trace of a “community of practice”^18^) that first appeared on the coasts of the Bismarck Archipelago 3535–3234 cal BP^19^. Lapita assemblages are marked by a variety of ceramic pot forms decorated with dentate stamped and other surface treatments, as well as obsidian tools, shell ornaments, and other items^20,21^. Once again, however, there has been a range of views; whilst many argue for a Lapita “package” brought by agriculturalists from ISEA^22^, without denying migration altogether, others have argued for “geographical mobility” over several centuries in the Bismarck Archipelago, rather than a directed demic diffusion.

The “out-of-Taiwan” model was driven primarily on proto-lexicon reconstructions by Blust^23^, with the archaeological and genetic evidence sometimes thought to have been moulded to fit this interpretation; and has been increasingly challenged from those disciplines^22^. For example, Cochrane et al. argued that the radiocarbon evidence for the spread of pottery in ISEA – thought by proponents of the “out-of-Taiwan” model to be the precursors for Lapita – does not support a directional dispersal from Taiwan towards Near Oceania, but rather multi-directional movements possibly centred on the Philippines and/or Borneo^24^.

The study of commensals has also pointed to a more complex picture. Domestic pigs in Remote Oceania trace back to mainland Southeast Asia, rather than ISEA/Taiwan, and the indigenous rats (*Rattus exulans*) to several sources in ISEA and the Western Pacific but are also absent from Taiwan^25^. Pacific dogs seem to share a common ancestry with Australian dingoes in the Yellow and Yangtze River basins of China^26^ but have not been found in the western Pacific of Lapita times^25^. On the other hand, the mtDNAs of domestic chickens in Remote Oceania nest within a subclade found in the Philippines, although a sister clade found across Indonesia is not seen in the Remote Pacific, and neither clade is found in Taiwan^27,28^.

Alternative models centre more on developments within Near Oceania, and stress spheres of interaction within Near Oceania and with ISEA^22^. For example, rising sea levels ~ 6 ka created coastlines across Near Oceania that might have facilitated substantial population movements^29^. Donohue and Denham^30^ and Torrence & Swadling^31^ argue that maritime interactions preceded and facilitated the spread of Austronesian languages, with the latter pointing to a rapid spread of Austronesian culture through pre-established networks between Biak Island, northwest of Papua, and Bougainville, west of the Solomon Islands (although see Spriggs^16)^. Zooarchaeology shows long-distance transport of forest wallabies from Sahul into ISEA before 12.8 ka and westward into the Maluku Islands^32^ evidencing New Guinea–centred exchange from the Terminal Pleistocene to the mid-Holocene, and genome-wide data from Wallacea has also recently indicated human movements in the same timeframe^33,34^.

Various models for the expansion of Lapita-bearing populations and their descendants consider demographic viability, directionality, migration speed, and cultural and environmental conditions at the destination^17,35,36^. Although the simplest model would be a wave of advance (WOA) ^37^ along the axis of the Solomon Islands, the absence of early Lapita sites between Bougainville and the Solomons gave rise to the leapfrog model, initially introduced by Sheppard and Walter^38^, with the Temotu region (Reef/Santa Cruz Islands) settled directly in a leapfrog movement by Austronesian-speaking people from the Bismarcks. Leapfrog models contrast with WOA models by bypassing regions with lower suitability for settlement in favor of areas with higher habitat quality and have recently been supported by genome-wide data^39^. At least from Samoa eastwards, genetic evidence clearly supports a sequential migration with progressive loss of diversity^40^.

Equipped with wooden voyaging canoes and outriggers, Lapita populations expanded rapidly into parts of Remote Oceania, with confirmed settlements in Reef/Santa Cruz, Vanuatu, New Caledonia, Fiji, Tonga, and Samoa. Here we define Near Oceania as encompassing mainland New Guinea, New Ireland, New Britain, the western Solomon Islands, and adjacent islands, while Remote Oceania includes all archipelagos beyond this boundary. Within the Solomon Islands, the earliest deposits date to 3.2 ka, with fast colonization of Reef/Santa Cruz Islands between 3.2 and 3 ka^41^, and with Vanuatu at the same time^42,43^. Other regions of Remote Oceania, such as the Mariana Islands and Palau, may have been settled coevally but by culturally distinct populations with shared deeper ancestry. The rest of Remote Oceania was colonized in succession in the following millennia, although the Lapita complex itself did not progress beyond Tonga and Samoa and disappeared by ~ 2.7 ka^44^. Spriggs speculated that this may have been due to the impact of Papuan cultures with a more egalitarian ideology diffusing east in the wake of Lapita^45^, a possibility recently strengthened by ancient DNA (aDNA) evidence^46^. Aotearoa/New Zealand to the south, with a robust colonization chronology, was one of the last regions to be colonized at ~ 750 BP^47–50^.

Ancient DNA studies have recently leant support to a model in which people carrying the Lapita complex initially moved from the Bismarck Archipelago to Remote Oceania with minimal admixture with local populations^46,51–53^. However, while the colonization of the western Pacific by bearers of the Lapita complex is not contested by genetics^40,54^, the deeper, “ultimately East Asian”^46^ ancestry of the “First Remote Oceanian” populations remains contentious. The mitochondrial haplogroup B4a1a1a–16,247, a maternal subclade widely distributed at very high frequencies across Remote Oceania – and therefore labelled the “Polynesian motif” (PM) – has been pivotal to the debate. Although labelled “Polynesian,” this is strictly speaking a misnomer because the PM predates the settlement of Polynesia and has a geographically broader distribution^55^.

Many authors attribute its presence to a mid-Holocene dispersal from East Asia, in accordance with the “out-of-Taiwan” model^56–61^. However, Brandão found that only ~ 20% of the maternal ISEA genetic pool – crucially, not including the PM – had a mid-Holocene Taiwanese ancestry^62^, supporting arguments for an earlier origin of the PM, either in Eastern Indonesia^63–65^ or in Near Oceania^54^. Since haplogroup B4 originated on the Asian mainland^66,67^, this would point to the spread of an East Asian genetic component through ISEA and into the western Pacific in the late Pleistocene/early Holocene^68,69^, rather than the mid to late Holocene.

The analysis of Y-chromosome diversity has also suggested that today’s Oceanic speakers have a primarily maritime heritage^70–73^, suggesting to some authors a sex bias during a mid-Holocene “out-of-Taiwan” dispersal, with dispersing women from the Asian mainland assimilating local males en route through Southeast Asia and Near Oceania^56,71–73^. Recent high-profile aDNA analyses suggest, instead, that the sex bias arose after Lapita settlement in the Western Pacific^51–53^. However, although these recent aDNA analyses of Lapita burials from Vanuatu and Tonga were interpreted by their authors as strongly supporting the mid-Holocene “out-of-Taiwan” model^51–53,74^, it is important to note that those analyses did not distinguish between a mid-Holocene and a late Pleistocene/early Holocene ancestry, and therefore were unable to rule out the latter alternative hypothesis.

The use of phylogeographic analysis, with a focus on haploid markers and molecular clocks, allows for the estimation of lineage ages and dispersal timelines not currently possible with autosomal data and where critical aDNA is lacking^75,76^. Two very similar mtDNA mutation rates have been widely used in the last decade. The time-dependent mutation rate of Soares et al.^77^ was obtained from paleontological evidence for the human–chimpanzee split and adjusted for the effect of purifying selection at recent time depths, whereas the rate of Fu et al.^78^ and its successors were obtained using a Bayesian analysis of dated aDNA sequences. For recent time intervals, the rates differ by only ~ 10%, so the differences are not critical for events taking place mostly in the last 5 ka or so. Nevertheless, given the small branch lengths analyzed, rates based on aDNA can be easily overestimated due to only one or two artefacts (i.e., sequencing errors, which often occur in aDNA sequences^79^, reflected in the very large confidence intervals of the Fu et al. rate, so that it overlaps with the Soares et al. rate in this range. For this reason, we employ the Soares et al. rate here. Also, the combination of the founder analysis methodology and the Soares et al. rate already provided reliable migration estimates in other parts of the world, such as the Holocene period in Africa^80^. The main justification for the use of this particular rate, however, is that it yields dates in the current work that closely match the radiocarbon evidence for the settlement of Remote Oceania, as we demonstrate below.

We collected the largest dataset of PM mitogenomes yet assembled: 1364 PM mitogenomes, including 234 that were new to this study (blood samples collected with individual-level consent and full ethical approval), to investigate the context and deep ancestry of Pacific colonization. Our results show that genetic founder estimates for island colonization closely match radiocarbon dates, validating the molecular clock methodology employed. This strong support for the molecular clock enables us to then infer that the PM likely arose along the northern coasts of New Guinea at least 6 ka, predating the hypothesized mid-Holocene Austronesian expansion, and providing a refined framework for understanding the early spread of Oceanic populations. These findings support a scenario in which technological and behavioral shifts, together with established interaction networks and potential environmental influences, likely contributed to the emergence of the Lapita complex and the subsequent settlement of Remote Oceania.

## Materials and methods

### Population sampling

DNA samples from PNG were collected with individual named consent by Stephen Oppenheimer and George Koki: see Bergström et al.^81^. Samples from Vanuatu were originally collected with individual-level consent in multiple local sub-studies and updated approval for sample use has been received from the Vanuatu Cultural Centre. These, and other samples from the Solomon Islands were collected as part of multiple projects performed between 1980 and 2000 with individual-level consent, coordinated by John Clegg and others, and now stored as part of the Oceanian Genome Variation Project (OGVP), with approval for use by the Oxford Tropical Research Ethics Committee. All OGVP samples are now fully anonymised. Previously unpublished University of Huddersfield mitogenome data include one Malay mitogenome from a sample collected by Zafarina Zainuddin, with individual named consent, approved by Human Research Ethics Committee, Universiti Sains Malaysia and the University of Leeds Faculty of Biological Sciences Research Ethics Committee. The DNA sequencing was approved by the Ethics Committees of the University of Huddersfield, School of Applied Sciences. All methods were performed in accordance with the relevant guidelines and regulations approved by the Ethics Committees.

**Fig. 1:**
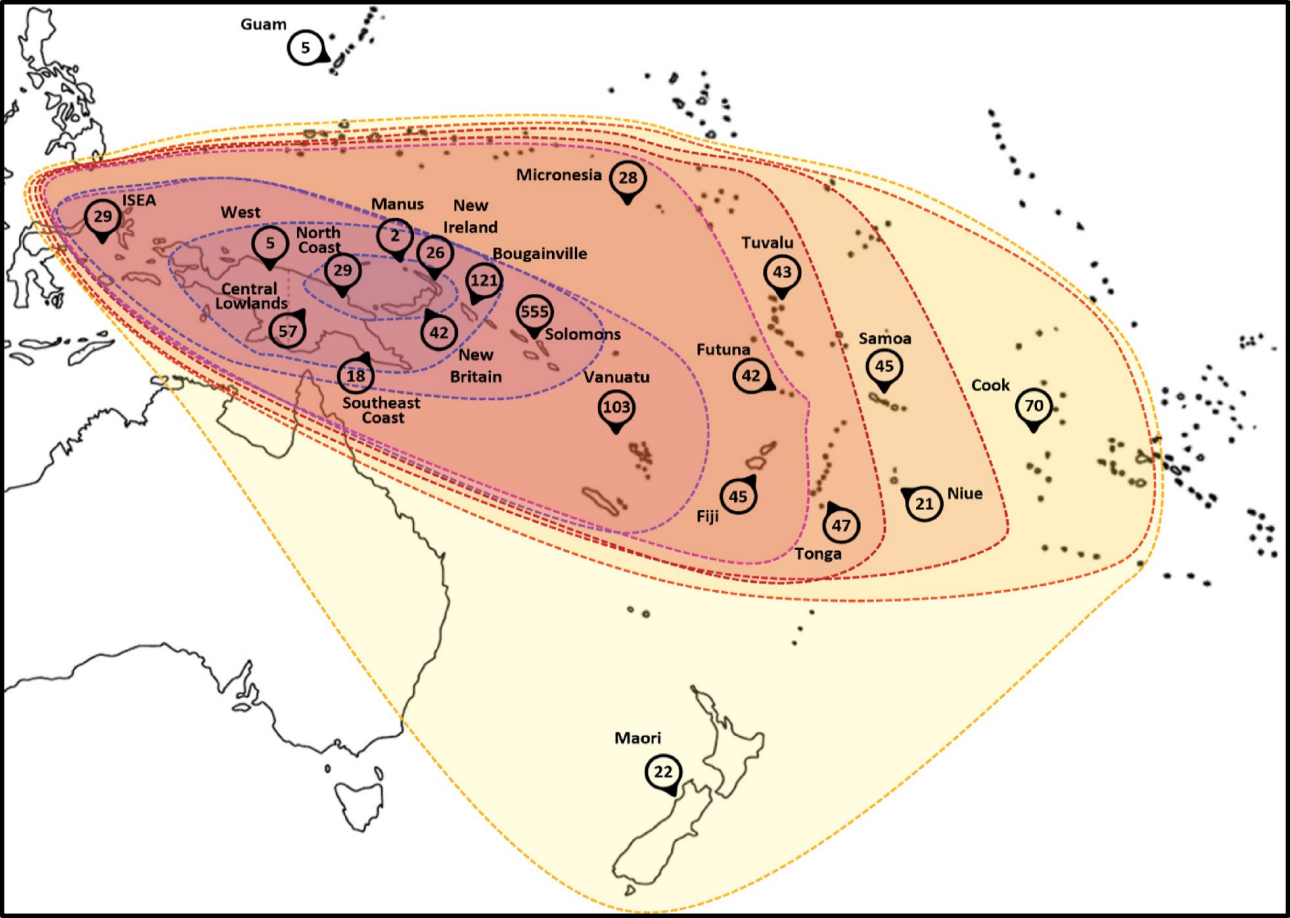
Map of sampling sites from Island Southeast Asia to Remote Oceania and the defined source and sink regions for the founder analysis. Numbers indicate sample size for each location, and we used islands located within each contour as sources for the sinks in the next larger region. Novel samples were retrieved from North New Guinea (one sample), Lowland New Guinea (seventy-four samples), southeast coast New Guinea (two samples), New Britain (one sample), New Ireland (one sample), Manus (two samples), Solomons (nineteen samples), Guam (three samples), Kiribati (six samples), Nauru (eighteen samples), Vanuatu (ninety-one samples), Fiji (eight samples), Cook Islands (seven samples). Additional locations, not shown in the map, include Malaysia (two samples – one of them novel), Hawai’i (three samples), Madagascar (three samples) and Somalia (one sample). Contour map modified from vecteezy.com under copyright free Creative Commons.

We compiled a comprehensive dataset comprising 1364 samples derived from almost 70 different islands from ISEA to Near and Remote Oceanians (Fig. 1). This dataset included both published (NCBI and 1000 Genome Project), including 2 ancient Māori samples from New Zealand, and 234 new mitogenomes (see Supplementary Tables 1 and 2), which we either amplified using 32 overlapping fragments with primers and PCR conditions as described before following Sanger sequencing with the same primers on a 3100 DNA Analyzer (AB Applied Biosystems)^54^, or following amplification of two large overlapping fragments and sequencing with Illumina MiSeq paired-end sequencing (fragment size 150 bp) (Earlham Institute, Norwich Science Park, UK), as previously described^82^. We analysed the Sanger sequences with SeqScape (AB Applied Biosystems) and BioEdit version 7.0.4.1^83^. We employed EAGER pipeline^84^ for initial processing, including quality assessment with FastQC^85^ and read preparation with AdapterRemoval v.2.2^86^. We aligned the reads to rCRS using BWA-MEM^87^, identified PCR duplicates with DeDup^84^, and performed quality control with QualiMap v.2.2.1^88^. We used GATK v.3.7-0-gcfedb67^89^ for realignment and variant calling and SNP filtering with VCFtools v.0.1.11^90^ was based on quality (Q30) and coverage (5x) criteria. Further filtering with BCFtools v.1.4^91^ and shell commands isolated polymorphisms with allele frequencies above 0.70, excluding those below 0.30, and flagged potential heteroplasmic positions for manual inspection in IGV v.2.323^92^. We assigned the samples to their respective haplogroups using HaploGrep 2.021^93^. We selected unpublished samples from the deposited complete mtDNA genomes in GenBank after identification of B4a1a1 samples using HaploGrep 2.021^93^. We excluded sequences with excessive ambiguous sites or length anomalies.

### Phylogenetic analysis

We constructed a phylogenetic tree using a reduced-median algorithm^94^ with Network 5 and visualized in Network Publisher 1.3.0.0 (Fluxus Technology Ltd). To ensure an accurate representation and minimize excessive reticulation, we down-weighted mutations with high homoplasy (146, 150, 152, 195, 16093, 16129, 16189, 16311 and 16362) from the default value of 10 to 7. We also removed positions reported as inconsistent (308–315, 310 C, 3107 N, 515–522, 16182 C, 16183 C, 16192 C, 16518 T and 16519 C). For inferring the most plausible tree structure, we combined the online mitogenome tree (PhyloTree Build 17^95^) along with mutation rates for each position^77^. This way, the tree is built prioritizing branches established by less frequent mutations. When reticulations involved polymorphisms with equal probabilities, we considered factors such as clade diversity and number of individuals within each node. We reconstructed a tree in Microsoft Excel displaying all samples and mutations involved for each clade (Supplementary Table 3).

### Data analysis

We sub-divided the dataset according to the geography as displayed in Fig. 1 and calculated basic diversity measures such as nucleotide diversity (π), using the (DnaSP) v6 software (http://www.ub.edu/dnasp/)^96,97^, and the *ρ* statistic^98^ for each region, excluding locations with less than 15 samples. We focused on B4a1a1–14,022^54^, because the A-to-G transition at position 16,247, which traditionally defined haplogroup B4a1a1a (when only the mtDNA control region could be analysed), has been shown to be highly variable in this haplogroup, with multiple independent back mutations^99^. We confirmed this feature across various independent datasets. Given the similar distribution of B4a1a1 and B4a1a1a, we therefore here combined the two into one analysis.

We reviewed the literature for radiocarbon estimates, including 2σ confidence intervals for Lapita colonization of the major islands across Near and Remote Oceania, enabling a collection of archaeological information from across the Pacific. For Near Oceania, settled in the Pleistocene, we focused on the emergence of Lapita pottery. For Remote Oceania (where Lapita ware did not spread beyond Tonga and Samoa), we collected ages of first settlement.

Surfer v8 software (https://surfer.software.informer.com/8.0/) enabled us to visualize both genetic and archaeological results in a spatial format based on interpolations based on a limited amount of datapoints. This software uses the coordinates of each island of interest alongside its corresponding data point (archaeological date of arrival, estimated genetic time of arrival or genetic diversity). Surfer displays data as a gradient format with putative source regions (higher values) displaying darker colours and sink regions resulting from the migration wave with lighter shades. Interpolation was based on the Kriging software^100^. For specific islands, where both archaeological and genetic data were available, we then plotted one against the other, in order to draw a direct comparison between them.

Despite the high diversity of the Polynesian motif within the Bismarck Sea more generally, its low diversity in New Britain is noteworthy. However, New Britain shows a highly unusual pattern of haplotype diversity, with various derived haplotypes at high frequency (rather than the root type of the motif, as in most other Pacific populations). This is a feature of populations that have experienced heavy genetic drift – it is particularly evident, for example, in the Anem group from Duggan et al.^101^ Given this pattern, we opted for removing these data from the interpolation map.

### Founder analysis

By defining a specific migratory event from a source region, and assessing the diversity of the source, founder analysis uses the newly accumulated genetic diversity within the settlement zone (sink) to estimate genetic settlement times^80,102,103^. We used geographic criteria to classify samples as either source or sink, assuming a largely west to east expansion, based on the archaeological data. Following an assessment of diversity for a putative source location, we evaluated founder ages successively from the Bismarck Archipelago and north coast of New Guinea, taking into account for each successively defined area, comprising one or more sink populations, all the diversity within the previously defined area as the source, as displayed in Fig. 1. The phylogenetic reconstructions enabled us to identify variants that were most likely already present in the source population and carried from there into the sink population. This allowed us to consider only the private mutations that arose in the settled region, providing an estimate of the time of migration by using the molecular clock. Thus, founder ages correspond to an overall *ρ* estimate of all the private diversity in the region. Age estimates are then derived solely from mtDNA variation without incorporating archaeological dates or other external chronological constraints: they are fully independent.

We calculated the estimated arrival time of a subclade into a sink region with the *ρ* statistic^98^, with standard deviations estimated as previously^104^. Despite some erroneous critiques, the *ρ* statistic is an unbiased estimator that is independent of the tree topology^105^. As sample sizes are not very large for each founder cluster, we opted to calculate an average *ρ* based on the accumulated diversity in each distinct founder cluster for each region. We corrected the estimates for the effects of purifying selection using the time-dependent molecular clock for complete mitogenomes^77^. All genetic estimates are displayed in Supplementary Table 4.

### Statistics and reproducibility

All statistics analyses, including nucleotide diversity (π)^96,97^, and the *ρ* statistic^98^ follow standard, widely accepted protocols and are fully reproducible with the available data. Every geographical subset used in the analyses is explicitly defined in both the main text and the Supplementary Material, ensuring complete transparency and replicability.

## Results

**Fig. 2:**
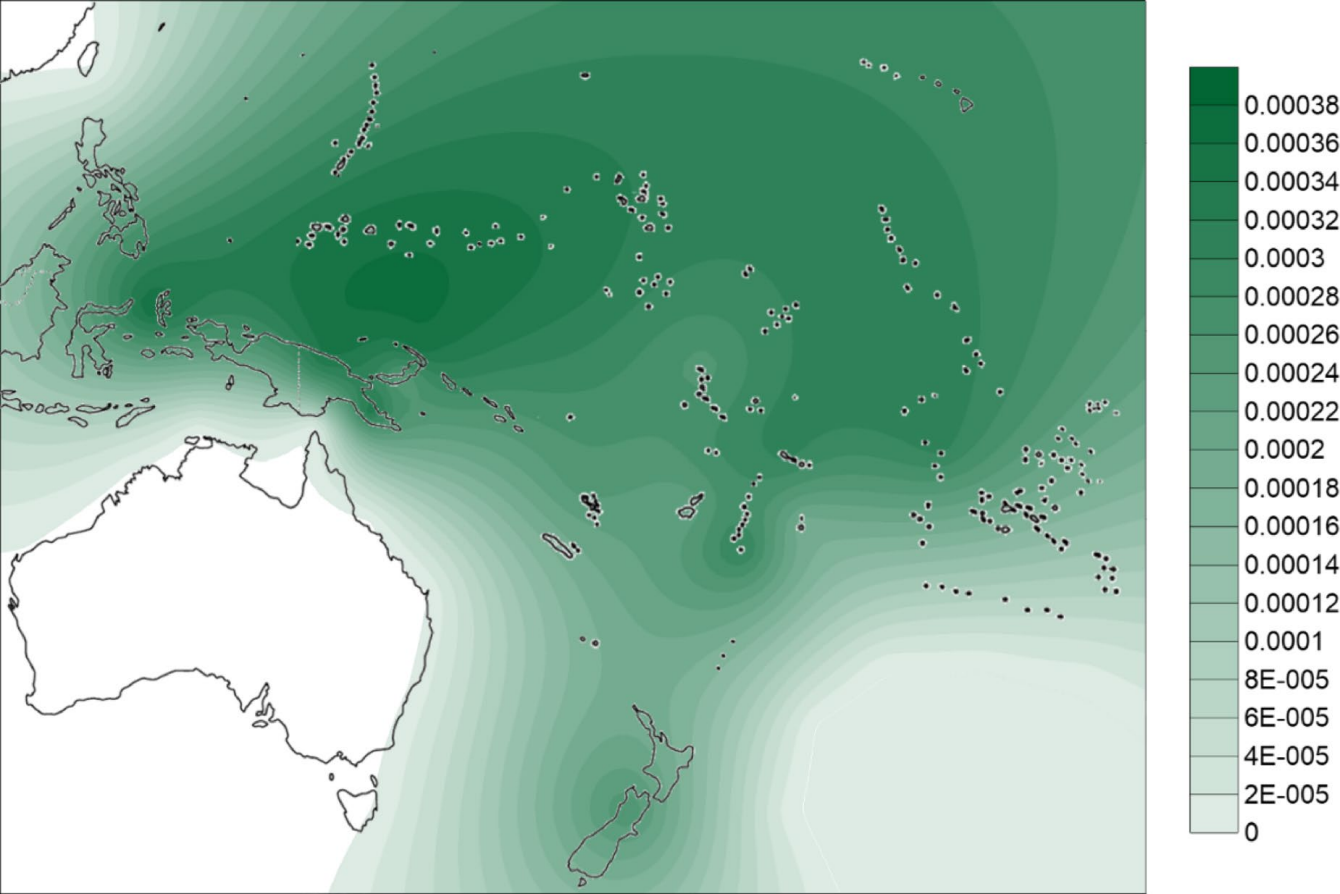
Distribution of nucleotide diversity for mtDNA haplogroup B4a1a1 using DnaSP 6. Interpolation was obtained with the Kriging algorithm in Surfer 8 software. Contour map modified from vecteezy.com under copyright free Creative Commons.

**Fig. 3:**
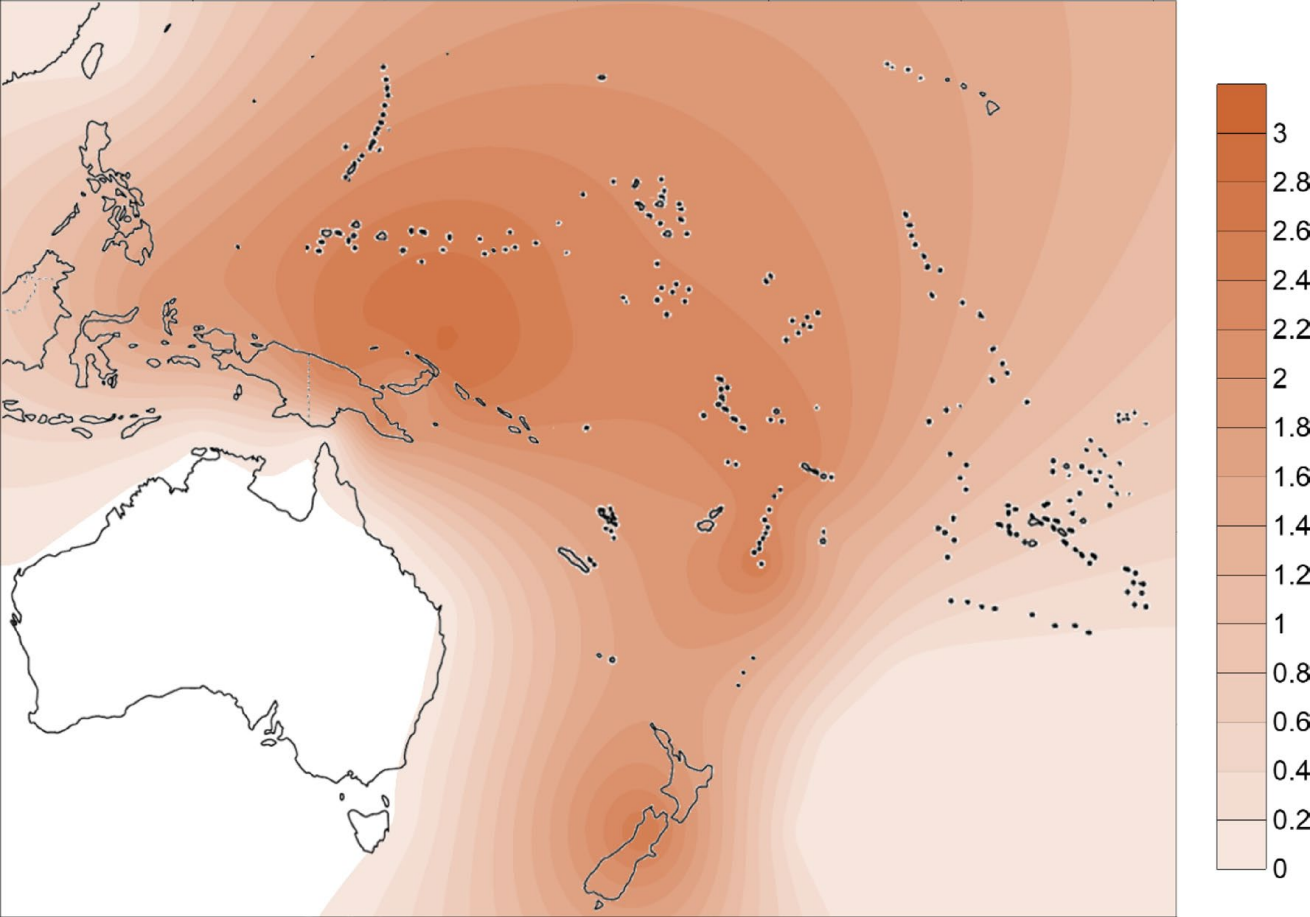
Distribution of *ρ* statistics for mtDNA haplogroup B4a1a1. Interpolation was obtained with the Kriging algorithm in Surfer 8 software. Contour map modified from vecteezy.com under copyright free Creative Commons.

From the phylogenetic reconstruction, we obtained a comprehensive tree of mtDNA haplogroup B4a1a1. We calculated diversity measures with DnaSP 6 and the *ρ* statistic (using Network)^105^, as shown in Table 1. We geographically interpolated the diversity measures using the Kriging algorithm (Figs. 2 and 3) and, broadly, diversity was highest on the north coast of New Guinea, New Ireland and Bougainville with the highest diversity point located around the Bismarck Sea.

**Table 1:** Diversity measures for each location assessed, based on π (nucleotide diversity) and *ρ*.

Islands	Diversity	
	π	ρ
ISEA	0.00036	1.966
PNG Central Lowlands	0.00028	2.123
PNG North Coast	0.00034	2.464
PNG Southeast Coast	0.00034	2.389
New Britain	0.00023	1.643
New Ireland	0.00035	2.769
Bougainville	0.0003	2.603
Solomon Islands	0.00026	2.268
Micronesia	0.00033	2.250
Vanuatu	0.00025	1.854
Fiji	0.00022	1.933
Futuna	0.00028	2.286
Tuvalu	0.00025	2.256
Samoa	0.00031	2.333
Tonga	0.00031	2.596
Niue	0.00019	1.524
Cook Islands	0.00031	1.214
Aotearoa/New Zealand	0.00026	2.650

**Fig. 4:**
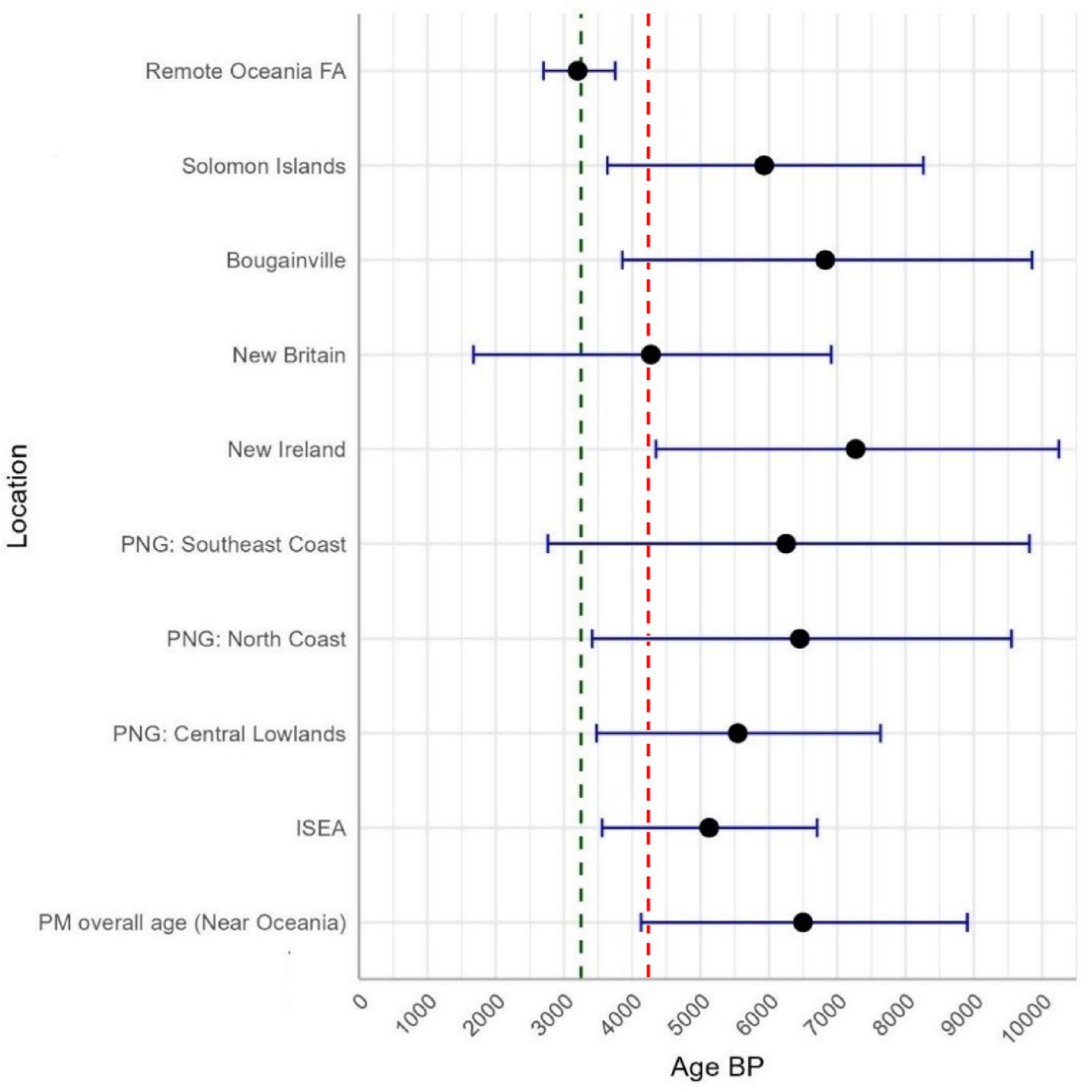
Age estimates with 95% confidence range of the PM overall in Near Oceania, and for each potential source region in Near Oceania, as well as Wallacea, with the founder age (FA) in Remote Oceania for comparison (using the two most diverse regions, PNG north coast and New Ireland, as source). Dashed lines show the putative archaeological arrival of the Neolithic/Austronesian speakers in ISEA (red) and the Bismarck Archipelago (green).

We further scrutinized diversity by considering the age estimates based on *ρ* across regions, to investigate potential points of origin: effectively, any point within the distribution of B4a1a1 outside Remote Oceania. We show age estimates based on *ρ* and a time-dependent molecular clock^77^ in Fig. 4. North New Guinea (PNG north coast), New Ireland and Bougainville display the oldest PM age estimates, all predating a possible late Holocene arrival in the region from Taiwan, even when considering the 95% confidence intervals. This is also true for ISEA, although the *ρ* diversity is lower than most estimates for Near Oceania. Only the lower bounds of our estimates reach the putative arrival of the haplogroup in ISEA (dashed red line: 4.2 ka^11^) or in the Bismarcks (dashed green line: 3.25 ka) under the “out-of-Taiwan” model and, for New Ireland, Bougainville and the Solomons the lower bounds substantially predate the latter. New Britain is exceptional in displaying a low genetic diversity, suggesting high genetic drift as we will discuss later. This pattern is also reflected in the very low nucleotide diversity (0.00023) in Table 1, the lowest diversity for the PM outside Remote Oceania.

The genetic data suggest that the PM arose (by mutation, from its immediate B4a1a ancestor) ~ 6.5 ka in the vicinity of the Bismarck Sea, with subsequent dispersals to the west, north and south – but mostly to the east, towards Remote Oceania. We inferred a combined north New Guinea/New Ireland origin of the PM – broadly, the primary ancestral source region for Remote Pacific colonization – and used founder analysis to estimate arrival times, by subtracting the source diversity from the estimates. Before considering a more detailed model, we estimated an overall founder age for the settlement of Remote Oceania at 3.2 ka (95% CI: 2.7–3.7 ka), consistent with archaeological evidence for the initial settlement of Vanuatu, regarded as the first stage in the colonization of Remote Oceania^43^.

**Fig. 5:**
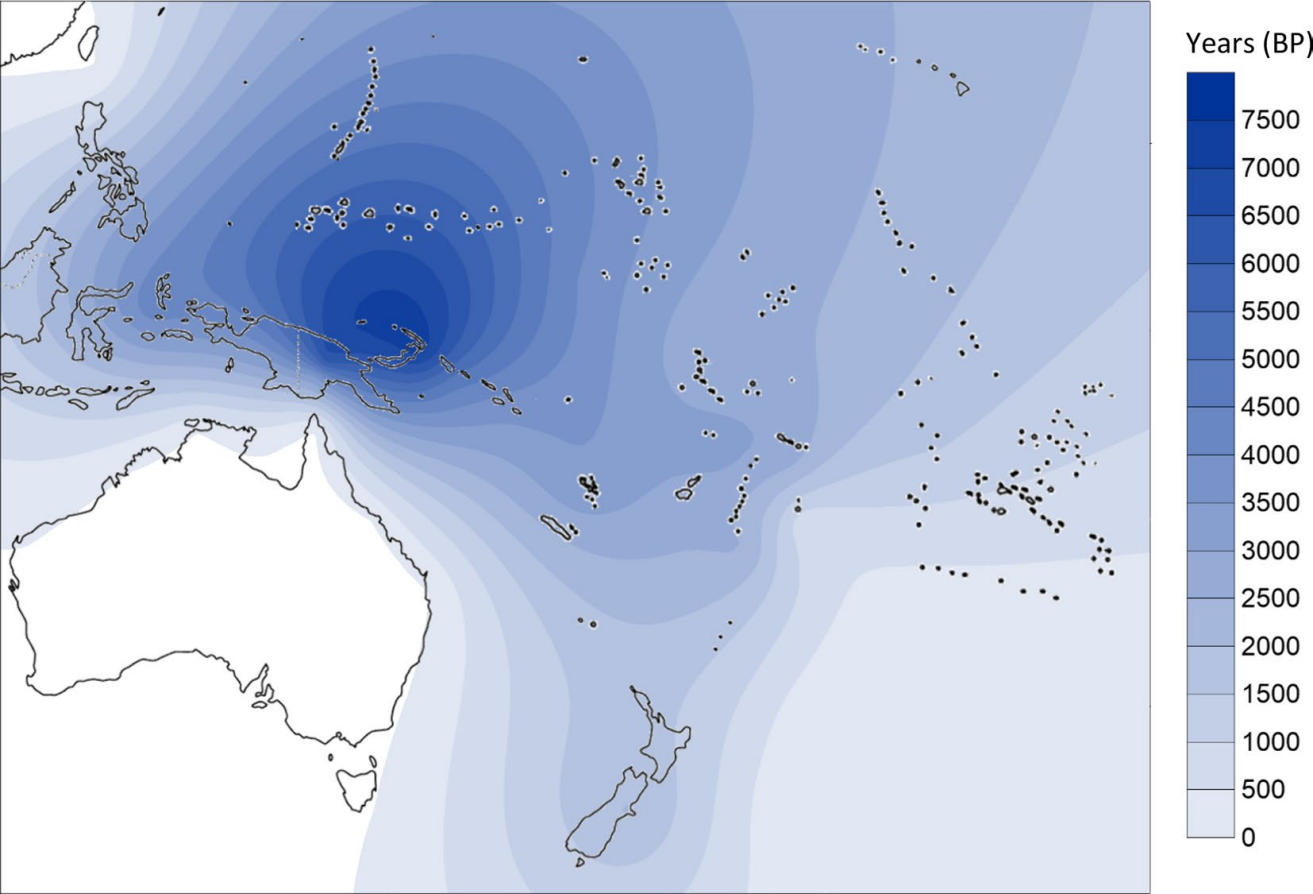
Spatial distribution map of the origin and expansion of the Polynesian motif. North New Guinea and New Ireland display age estimates whereas the remaining values are founder ages. Image obtained with the Kriging algorithm in Surfer 8 software. Contour map modified from vecteezy.com under copyright free Creative Commons.

**Fig. 6:**
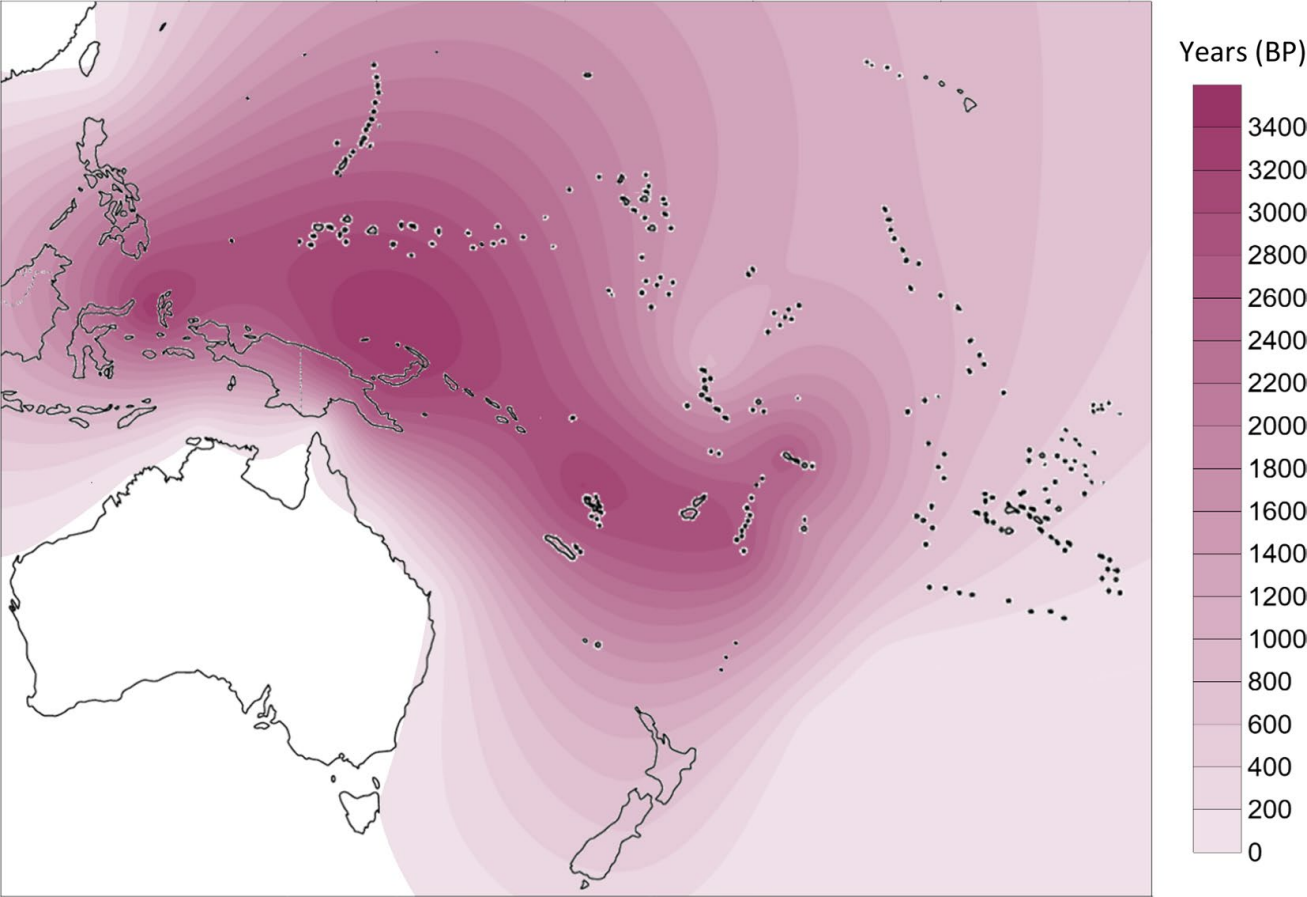
Spatial distribution map of early Lapita and Remote Pacific colonization according to archaeological remains. The first Lapita deposits are located at the Bismarcks, where the culture most likely arose^44^. We did not include “frontier Lapita” data^106^. Image obtained with the Kriging algorithm in Surfer 8 software. Contour map modified from vecteezy.com under copyright free Creative Commons.

We further estimated genetic founder ages of ~ 4 ka near the boundary between Near and Remote Oceania, and ages of ~ 3 ka and below for islands in Remote Oceania (Table 2). We compared the results with archaeological estimates of the appearance of Lapita or (beyond Tonga/Samoa) first settlement in different locations (Table 2) and displayed the results as geographic interpolations respectively for genetic and archaeological data (Figs. 5 and 6). Table 2 presents both the genetic and archaeological age estimates for the settlement of different islands, together with the relevant literature sources for the dating evidence. For Micronesia it would be difficult to correlate the genetic founder age of the PM with a specific age of settlement without further assumptions, and we therefore just present the genetic estimate.

**Table 2:** Comparison of Polynesian motif (PM) founder estimates, based on *ρ*, and archaeological data for appearance of (1) Lapita, (2) “frontier Lapita”^106^, (3) red-slipped pottery in Moluccas, or (4) first settlement, post-Lapita. North new Guinea and new Ireland are considered as the source region for the PM, and therefore we show age estimates and not founder ages for these, and we also give an overall age estimate for the PM (estimated from all near Oceania data, excluding new Britain). Note that the dates for “frontier Lapita,” lacking Lapita ceramics, in Southeast new Guinea and the Solomons are closer to the ages of the PM than of Lapita proper.

Location (*n* for genetic data)	Age estimate (BP)		Archaeological source
	Genetic founder age with 95% confidence range	Archaeological C14 age, 95.4% (cal BP)	
Wallacea (32)	4300 [2890–5740]	3740–3020^3^	Cochrane et al.^24^
PNG Central Lowlands (80)	3550 [2600–4510]	–	–
PNG North Coast (15)	6450 [3410–9550]	3300–2700^1^	Terrell and Schechter^107^
PNG Southeast Coast (9)	3460 [1900–5030]	3480–3060^2^	Shaw et al.^106^
PNG Southeast Coast (9)	3460 [1900–5030]	2900–2500^1^	David et al.^108^
PNG New Ireland (26)	7270 [4350–10250]	3580–3180^1^	Spriggs^15^
PNG Bougainville (121)	4820 [3130–6530]	–	–
Solomon Islands (555)	3700 [2960–4450]	4080–3150^2^(Nissan)	Shaw et al.^106^
Solomon Islands (555)	3700 [2960–4450]	3185–2639^1^	Sheppard et al.^109^
Micronesia (33)	3900 [1600–6230]	–	–
Vanuatu (103)	3450 [2490–4410]	3430–3030^1^	Denham et al.^43^
Fiji (45)	2590 [1610–3570]	3050–2950^1^	Clark & Anderson^110^
Futuna (42)	3270 [1630–4930]	2300–2000^4^	Kirch^111^
Tuvalu (43)	2460 [1310–3630]	1070–770^4^	Dickinson et al.^112^
Samoa (45)	2880 [1980–3770]	2900–2600^1^	Cochrane et al.^113^
Tonga (47)	2530 [1680–3380]	2846–2830^1^	Burley et al.^114^
Niue (21)	610 [0–1250]	2000–1600^4^	Walter and Anderson^115^
Cook Islands (70)	1510 [850–2170]	1150–725^4^	Sear et al.^35^
Aotearoa/New Zealand (22)	2060 [770–3370]	700–675^4^	Bunbury et al.^47^
Polynesian motif overall (Near Oceania) (806)	6499 [4128–8906]	–	–

From the compiled archaeological data, we built a spatial distribution map, shown in Fig. 6. The earliest known Lapita cultural deposits are in the Bismarcks, now thought to be as late as ~ 3.25 ka^19,44,116^, with recent researchers dismissing a posited Lapita source in the Marianas^19^, which were first settled at a similar date, likely from the Philippines^117^. However, the precise origins remain debated, with archaeological evidence^118^, sailing simulation models^119^, and genetic data^120–122^ pointing to different potential source regions. Lapita spread explosively across Near Oceania and into Remote Oceania as far as Tonga and Samoa within a few centuries of its appearance in the Bismarck Archipelago^44^. Eastward population movements later culminated with the exploration of East Polynesia around ~ 1000 cal BP^21,50^. The two maps (Figs. 5 and 6) highlight the similarities between genetic and archaeological pictures.

**Fig. 7:**
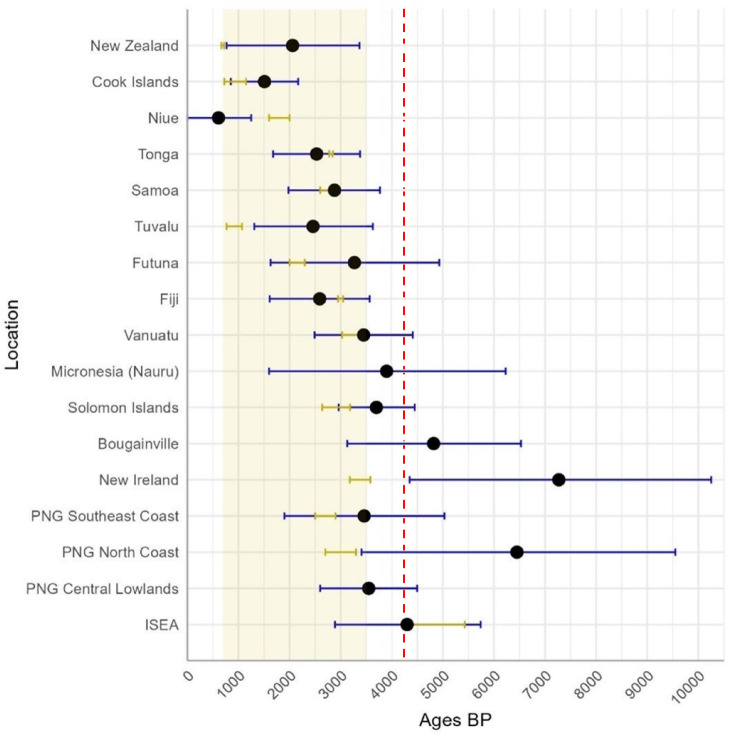
Comparison of archaeological (ochre) and molecular (blue) founder age estimates in Wallacea, Near Oceania and Remote Oceania, as per Table 2. We consider North New Guinea and New Ireland as the source region for the PM, as they are the regions with highest diversity, and therefore we show age estimates and not founder ages for these. Ochre shading indicates the approximate range of archaeological settlement times in Oceania, from Lapita onwards. Dashed red line shows the putative archaeological arrival of the Neolithic/Austronesian speakers in ISEA^11^.

To further compare archaeological and molecular data, we prepared a linear correlation between age estimates for islands with both age settlements estimates (Fig. 7). Colonization proceeded from west to east, with both lines of evidence for settlement of Remote Oceania in broad agreement. This alignment further supports the validity of the molecular clock we used.

The two sets of exceptions (where the 95% confidence interval does not include the archaeological estimate) are in Near Oceania, prior to the Lapita expansion, where the emergence of the motif predates the emergence of Lapita, and Niue, Tuvalu and Aotearoa/New Zealand, which we discuss below. Notably, the southeast coast of New Guinea and the Solomons correlate with the archaeology, suggesting the presence of the motif here is part of the Lapita expansion.

## Discussion

### Leveraging genetic dates in Remote Oceania as a calibration for the timescale in Near Oceania and ISEA

The demographic history of Holocene Southeast Asia and Near Oceania contrasts sharply with that of Remote Oceania. While the former is complex and often ambiguous, the latter is relatively straightforward. This contrast allows us to test controversial models proposed for the settlement of Southeast Asia/Near Oceania by first evaluating how well our methodologies – particularly molecular clock dating and founder analysis—perform in the clearer context of Remote Oceania. Here, we have estimated the ages of settlement of Remote Oceania independently with genetic data and then tested them against the results of archaeology and radiocarbon chronology.

Firstly, we obtained an estimated founder age of ~ 3.7 [3.0–4.5] ka for colonization of the Solomon Islands by the bearers of Polynesian motif lineages, overlapping radiocarbon evidence for the beginning of the Lapita diaspora from northern New Guinea/Bismarck Archipelago. Considering their geographical distribution, bypassing these islands without making stops appears challenging. Our genetic data show a level of private diversity within the Solomons that falls along the gradient between the Bismarck Archipelago and Remote Oceania, suggesting that Lapita voyagers likely spread *via* at least parts of the Solomon Islands into the Remote Pacific at the time of the major migration event, contradicting the leapfrog model, which proposes direct migration from the Bismarcks to Remote Oceania, bypassing the Solomon Islands. This model is based on archaeological and linguistic evidence – a lack of earliest Lapita ceramics and the present-day Austronesian language distribution^123^. One other possible line of evidence for the leapfrog model is that post-Lapita dispersals carrying Papuan ancestry from the Bismarcks appear to have bypassed certain intermediate regions, reaching more distant islands directly – a pattern consistent with leapfrogging movements^39,52^. However, this is relevant only to the later post-Lapita dispersals, rather than to the early spread of Lapita itself. Given this, Remote Oceanian first settlement *via* the Solomons would not necessarily contradict the autosomal/aDNA evidence and might be accommodated archaeologically by the idea of “frontier Lapita” without ceramics as seen in New Guinea^106^, consistent with the very early age estimates we see for B4a1a1a in the Solomons, and with language shift as Papuan populations moved in to (partly) replace the first settlers.

We estimated ages of ~ 3.5–2.6 ka for locations within Near Oceania and western Remote Oceania (such as the southern coast of New Guinea, the Highlands of New Guinea, the Solomons and Vanuatu), and ~ 3 ka and below in the remainder of Remote Oceania, which fits the broader picture of colonization in Remote Oceania^42,124^ and provides strong support for the phylogeographic methodology we have adopted. Somewhat higher ages in the Solomons (3.7 [3.0–4.5] ka) and especially Bougainville (4.8 [3.1–6.5] ka) might indicate some gene flow from the Bismarcks and New Guinea north coast before the major expansion of the Lapita people.

There are just five age estimates whose 95% confidence intervals are outside the archaeological age for Lapita archaeology or the first settlement evidence from the islands in Fig. 6: New Ireland and the north coast of New Guinea for Lapita archaeology, and Tuvalu, Aotearoa (New Zealand) and Niue for the evidence of first settlement. The high time depth of the Polynesian motif in New Ireland and the north coast of New Guinea indicates that the B4a1a1 lineage arose by mutation from its B4a1a ancestor in this region, ~ 6.5 ka. According to the archaeological evidence, people in the Bismarcks, and potentially also the north coast of New Guinea, developed the Lapita culture ~ 3.25 ka^44^. Thus, the phylogeographic pattern suggests that Lapita was developed amongst people in whose maternal ancestors the motif arose, ~ 3–4 ka, and whose deep maternal ancestry traced back to the Asian mainland during the Terminal Pleistocene. Technological and cultural innovations, particularly the adoption of improved sailing technology that prompted expansion in all directions, were likely due to contact *via* interaction networks with Austronesian speakers, already established in the region to the west into ISEA and east into Bougainville and the Solomons, from whom the local people carrying B4a1a1 putatively assimilated the Oceanic language.

Tuvalu, which comprises a small number of lightly inhabited Polynesian islands and atolls to the northwest of Samoa, displays a much higher level of private diversity than we would expect from the existing archaeological age estimate for its first settlement. However, rather strikingly, the age estimate from genetic data in Tuvalu (2.5 [1.3–3.6] ka) is perfectly in line with the settlement time of other islands across the same longitude as Fiji and Samoa (Fig. 1) and would fit very well with settlement soon after Fiji, Tonga and Samoa. This discrepancy in dates may therefore be due to the lack of detection of the first settlement in the rather insubstantial archaeological record for these islands^112^, highlighting a hypothesis for future archaeological testing.

In the case of Aotearoa (New Zealand), the situation is different, as these islands have one of the best-defined chronologies for settlement in Oceania^47^. We suggest two possible explanations for the discrepancy in this case. One is that we lack data from what may have been important stepping-stones between the available genetic source populations and Aotearoa, such as the Kermadec Islands, which could have been a direct source, although archaeological or oral historical information suggests settlement from Central East Polynesia (Cook Islands, Society Islands). There are no genetic data available within a radius of up to 2,000 km around New Zealand. Some diversity might have emerged in an intermediate source, some source diversity might have been lost due to partial resettlement^125^, and specific haplotypes might have increased in frequency due to founder effects when moving south into Aotearoa. Another strong possibility is that the data might contain some sequencing or transcription artefacts (i.e., artificially induced “mutations” introduced by error) that have raised the founder estimate. For example, the rare non-synonymous mutation 6261 A appears twice independently in two different subclades of the Aotearoa dataset of only 22 samples.

For the situation of Niue, where the founder age estimate, at ~ 700 years, is considerably lower than the estimated time of Polynesian settlement, the explanation seems clearer. While the first settlement dates to about 2 ka, it is thought that following a conflict with Tonga, 500 years ago, the Niue Islands were resettled from there^115,126^. Indeed, nearly 70% of the Niue lineages have direct matches in Tonga. A founder age between the two events may reflect the loss of diversity from Tonga during this resettlement.

### The origin of the Polynesian motif and its implications for the “out-of-Taiwan” model

The standard model for the expansion of Austronesian speakers into the Remote Pacific – the so-called “out-of-Taiwan” model – proposes a major, rapid migration from South China, *via* Taiwan (arriving at ~ 5 ka^7,8^), into ISEA ~ 4.2 ka^11^. From Near Oceania, this rapidly led to the colonization of western Remote Oceania after ~ 3 ka, with the emergence of the Lapita complex and sophisticated voyaging technology en route.

This model is primarily grounded in linguistics. Austronesian was the most widespread language family prior to the era of European expansion, spanning from Madagascar to Hawai’i, Rapa Nui (Easter Island) and Aotearoa (New Zealand). The archaeological picture is more complex, and as we argue from the mitogenome results presented here, there is little or no mid-Holocene Taiwanese maternal ancestry in Remote Pacific populations.

In genetics, the standard model has been typically expressed in terms of a simple contrast between “Asian” *versus* “Papuan,” with “Asian” genomes dispersing into Papuan territory in the mid-Holocene, but this model is too simplistic. Our analysis here suggests a model in which the mtDNA B4a1a1 “Polynesian motif” that is almost fixed across Remote Oceania does indeed have an ultimately Asian mainland source, distinct from indigenous Near Oceanian ancestry dating to the first settlement 50–60 ka, but in the Terminal Pleistocene rather than the mid-Holocene.

Haplogroup B arose in Asia ~ 50 ka^77^ and is the oldest mtDNA lineage known in East Asia^78^. Haplogroups B4, B4a and B4a1 arose on the East Asian mainland between ~ 45 and 20 ka^54^. But B4a1a* (i.e., excluding the derived B4a1a1, the Polynesian motif) has never been seen on the Asian mainland: it is almost entirely restricted to Taiwan, the Philippines and Indonesia/East Malaysia, and it dates to ~ 8.5 [6.7–10.3] ka^54^. Thus, it cannot track a mid-Holocene dispersal from South China.

Moreover, the Polynesian motif itself – the root of haplogroup B4a1a1 – has never been seen in Taiwan or the Philippines and is rare even in Indonesia. Its age has been debated for nearly three decades^54,58,59,62,65,69,127^, including some of the earliest work carried out with aDNA^128^ and recent studies of the settlement of Madagascar^129^.

The age estimates based on *ρ* calculated here suggest that it arose ~ 6.5 ka, and the distribution of its genetic diversity suggests that its origin was most likely between the northeastern coast of New Guinea, the Bismarck Archipelago and Bougainville. This points to the coast around the Bismarck Sea as the most likely source, ~ 3 ka before the putative time of the arrival of Austronesian migrants from Taiwan (Table 1). Although no data are currently available for Near Oceania, analysis of aDNA from a 7.5 ka Sulawesi sample suggests that some degree of Asian-related admixture may have occurred prior to the Austronesian expansion, and could plausibly have extended into Near Oceania, where the Asian-related mtDNA lineage B4a1a1 may have emerged^130^.

This does not mean that there were no mid-Holocene dispersals from South China/Taiwan. Previous work suggested that the Austronesian languages likely spread in ISEA in part due to dispersals from Taiwan involving other lineages, rather than B4a1a1^68^ – lineages which dwindled from around a fifth of total mtDNAs in Indonesia to almost zero in the Western Pacific.

Nevertheless, this implies that whilst a numerically minority dispersal drove the spread of the Austronesian languages from Taiwan, the more significant process in the Western Pacific was acculturation and language shift. Language and genetics finally became completely decoupled from Taiwanese lineages on the maternal line of descent (with the Austronesian languages now re-coupled to B4a1a1) during early Lapita times in Near Oceania.

However, this seems not to have been the case on the male line of descent. Whilst the oldest Lapita-associated mtDNA lineages in Vanuatu and Tonga belong to B4a1a1, the oldest Y-chromosome lineages (> 2.5 ka) belong to Y-chromosome haplogroup O1a-M119 (specifically O1a1a1a-F140 plus a few O1a2-M110 in Vanuatu), which likely dispersed from Taiwan. O1a1a1a is a major modern lineage, which dates to 6.5 ka in the YFull Y-chromosome tree (https://www.yfull.com/tree/O-M119/) and is nested by Chinese lineages as well as including modern Chinese, aboriginal Taiwanese, Filipino, Indonesian, Malaysian and Singaporean lineages (YFull), and is thus an excellent candidate for mediating the spread of Austronesian languages (see also ref.^71^). It is not seen in the modern Remote Pacific Y-chromosome database, but there is a single New Zealand lineage in the large O1a-M119 clade to which it belongs (YFull), which overall has a similar distribution centered on China, Taiwan and Island Southeast Asia, almost reaching fixation in several aboriginal Taiwanese groups^70^. O1a2-M110 is a more minor lineage which dates to 5.2 ka and is similarly found in modern China, Philippines and Singapore, as well as single individuals in New Zealand and Hawai’i, and indeed even Madagascar (YFull), which received an influx of Austronesian speakers from Borneo^131^. A founder analysis based on STR-defined networks suggested that O1a subclades were mostly Neolithic migrants from Taiwan^68^. However, as has been known for many years, these lineages are vanishingly rare in the Remote Pacific^56,70,71,132,133^. O1a2-M110 still occurs in the Admiralty Islands (northern Bismarcks) at ~ 18% but with widely varying frequencies from island to island^71^. The most common “Asian” lineage amongst Polynesians (at extremely variable levels) is O2-M122, a lineage commonly seen today on the Chinese mainland and present across MSEA and ISEA^56,70^. The STR-based founder analysis mentioned above placed the entrance of O2a1 by early Holocene^68^.

The predominant Remote Pacific Y-chromosome haplogroup today is C1b2a1-M208, which originates in coastal Near Oceania ~ 9.4 ka. Rather similarly to the emergence of B4a1a1 from B4a1a, C1b2a1-M208 descends directly from C1b2a-M38, which itself dates to ~ 18.5 ka and is widespread across southern ISEA, Wallacea, and coastal New Oceania; like the mtDNA B4a, the Y-chromosome C1b has Pleistocene mainland Asian ancestry. C1b2a-M38 is, however, likely to have originated within the New Guinea region – perhaps in the Bird’s Head of West Papua, where it remains at high frequency^134^. However, it is also found at appreciable frequencies in Madang, in the east of PNG^81^, and possibly it may have been replaced in the PNG Highlands during the Neolithic expansion that is thought to have dispersed the Trans-New Guinea language family^134^.

Unfortunately, there is no aDNA evidence for the timing of the switch from O1a lineages to C1b2a lineages in the Remote Pacific, although the deep Near Oceanian Y-chromosome lineages belonging to haplogroup M began to appear in Vanuatu within around 500 years of the first Lapita arrivals^53^ and haplogroup S by a few hundred years later^51^. C1b2a lineages have not yet been identified before ~ 500 years ago, in Vanuatu and French Polynesia^52^, but were presumably present in Western Polynesia before the settlement of Central East Polynesia after ~ 900 AD^50,135^ and most likely earlier given the early appearance of M and S.

All of this suggests that the earliest stage of Lapita emerged in a process of integration between largely male maritime migrants from China/Taiwan/ISEA and women on the north coast of New Guinea or in the Bismarcks. These women were part of a population that had lived there for several millennia at least, albeit with ultimately Southeast Asian (rather than Oceanian) ancestry. The two communities would be difficult to distinguish at the genome-wide level, since both had ancestry in Southeast Asia, but the uniparental markers – allowing the tracking of male and female ancestries – are sharply distinct and point to a sex bias that is almost a mirror image of models proposed in the past (see below). The subsequent switch to C1b2a1-M208 adds an intriguing further dimension to ongoing sex-biased social processes during the Austronesian dispersals that merits further study, when more aDNA data become available.

In short, our data do not imply that Lapita culture emerged without influence from Austronesian-speaking immigrants from ISEA – on the contrary, they support the possibility of direct influence from the northwest. Nor do they mean that the linguistic model of Austronesian origins in Taiwan is false. As well as the Y-chromosome evidence, migrants from Taiwan into ISEA are clearly detectable in the mtDNA phylogeography, amounting to ~ 20% of modern ISEA mtDNA variation^62,68^. However, various authors have stressed the importance of spheres of interaction and trading networks across the north coast of New Guinea and Eastern Indonesia^31,136^, and our results suggest that these may have had a significant role in transmitting ideas between Austronesian voyagers and local communities around the coasts of Near Oceania. Again, Spriggs^15,16^ has stressed the spread of new identities mediated by material culture and the new language spoken by dispersing elites, in a process akin to modern globalisation, rather than the simple farming/language model espoused by an earlier generation of “out-of-Taiwan” theorists. This also seems an attractive framework for interpreting the evidence we now have from genetics.

## Conclusions

Using a greatly enlarged dataset for the mtDNA Polynesian motif in Oceania, we have corroborated the molecular clock within the B4a1a1 lineage with refined genetic age estimates for the settlement of islands in Remote Oceania, showing that these estimates closely match radiocarbon dates for first arrival. This means that we can use the clock to reliably infer processes in less well understood contexts.

Our findings suggest that the Austronesian linguistic dispersal from Taiwan to Oceania did not drive a major genetic shift in Near Oceania on the female line of descent. This excludes the possibility of any substantial maternal ancestry from mid-Holocene China or Taiwan reaching the Remote Pacific. The Polynesian motif, the major maternally inherited lineage in the Remote Pacific, was already present in Near Oceania before the arrival of Austronesian speakers posited by the “out-of-Taiwan” model, and the emergence of Lapita, by at least 3000 years. It was “Asian” rather than “Melanesian” insofar as it resulted from an expansion of ancestry from the Asian mainland at the end of the Pleistocene^54^. As such, it was deeply divergent from the indigenous Near Oceanian ancestry in New Guinea, the Bismarcks and the Solomons that first reached the region at least 50,000 years ago.

We note that recent aDNA analyses^51–53,74^, as well as some analyses of modern genome-wide data^137^, assume that all “Asian” ancestry in Near Oceania arrived in the mid- to late Holocene. Their claims to support the standard “out-of-Taiwan” model therefore fail to distinguish early from mid/late Holocene ancestry. Nevertheless, aDNA data have decisively refuted the suggestion of major female-biased sex bias in the process of Austronesian expansion^56,71,72^, since they make clear that the influx of ancient Near Oceanian Y-chromosome lineages took place after the initial Lapita dispersals.

Although our mtDNA results only explain maternal ancestry, other lines of evidence support a minimal role for mid-Holocene Taiwanese ancestry in the Remote Pacific. For example, Capelli et al.^70^, Karafet et al.^138^, and Kayser et al.^56,71,72,132,133^ showed that present-day Polynesians mainly carry three Y-chromosome haplogroups linked to Near Oceania – although aDNA suggests that these lineages may have replaced Chinese/Taiwanese male lineages post-Lapita (see above). Soares et al.^68^, analysing both uniparental systems alongside genome-wide data, found support for small-scale mid-Holocene migrations from mainland East Asia into ISEA, *via* both Taiwan and Mainland Southeast Asia (MSEA). Therefore, small-scale dispersals may have spread Austronesian languages south in the late Holocene, but without any major genetic transformation of the existing populations in Near Oceania. Long-established interaction networks between Near Oceania and ISEA may have mediated this linguistic, cultural and technological diffusion^31,139,140^, perhaps with the spread of new identities mediated by material culture and the new language spoken by the dispersing elite groups^15,16^, creating the conditions for the formation of the Lapita complex in Near Oceania and the subsequent major dispersals.

Culture, genetics and language rarely travel as a package; nor, indeed, do all parts of the genome. The Oceanic languages came later to the groups who spread the Lapita complex after ~ 3.5 ka, but their maternal genetic ancestry largely had deeper roots in the early Holocene of Near Oceania. The movement of Austronesian languages through the Indo-Malaysian archipelago to the edge of New Guinea was indeed mediated in part by dispersals from Taiwan^62,68^. However, the formation of Lapita, leading to the subsequent spread of the Oceanic branch of those languages across the Pacific, was a complex result of socio-cultural processes likely involving language shift from incoming males to local women. The subsequent genetic impact from Taiwan across Remote Oceania was much smaller than commonly appreciated.

## Supplementary Information

Below is the link to the electronic supplementary material.

Supplementary Material 1

Supplementary Material 2

## Data Availability

All novel human mitogenomes were submitted to GenBank with accession numbers PV740830 - PV741063 and are accessible at NCBI Nucleotide via this direct links www.ncbi.nlm.nih.gov/nuccore/PV740830 - www.ncbi.nlm.nih.gov/nuccore/PV741063.
