# Supplementary material for: Leveraging known Pacific colonisation times to test models for the ancestry of Southeast Asians
Source: Sci Rep. 2025 Oct 23;15:37044. doi: 10.1038/s41598-025-20856-3 (PMC12550076; doi:10.1038/s41598-025-20856-3)
Supplement: Supplementary file 1 — Supplementary Material 1 [file 41598_2025_20856_MOESM1_ESM.pdf]

Supplementary Table 1. List of sample size per location and their source.

	Jinam et al. (2012)	Purnomo et al. (2021)	Pedro et al. (2020)	Tabbada et al. (2010)	Soares et al. (2011)	Delfin et al. (2013)	Duggan and Stoneking (2013)	Ingman et al. (2000)	Ingman and Gyllensten (2003)	Pierson et al. (2006)	Duggan et al. (2014)	Hartmann et al. (2008)	Macaulay et al. (2005)	Lippold et al. (2014)	Reiff et al. (2011)	Corser et al. (2012)	Behar et al. (2012)	Family Tree DNA	Benton et al. (2012)	Knapp et al. (2012)	Razafindrazaka et al. (2010)	Brucato et al. (2019)	Novel samples	Total number of samples
Malaysia	1																						1	2
Island Southeast Asia		16		1	11	1																		29
West New Guinea		4			1																			5
PNG: North Coast		2	1		7		1		4														1	16
PNG: Central Lowlands			5																				74	79
PNG: Southeast Coast			6							1													2	9
Bismarck Archipelago: New Britain			3								38												1	42
Bismarck Archipelago: New Ireland			2		23																		1	26
Manus																							2	2
Bougainville			5								109	2	1	4										121
Solomon Islands							524		12														19	555
Guam															2								3	5
Micronesia										3						1							24	28
Vanuatu					11					1													91	103
Fiji											37												8	45
Futuna											42													42
Tuvalu											43													43
Samoa								1	2		42													45
Tonga									1	1	45													47
Niue											21													21
Cook Islands									2		61												7	70
Hawai'i																	2	1						3
Aotearoa: Maori																			20	2				22
Africa: Madagascar, Somalia																					3	1		4
																							234	1364

Supplementary Table 2. List of the 234 novel mitogenomes used in this paper, as well as sample size for each location. The colour code is the same used in Supplementary Table 3.

Sample ID	GenBank accession number	Location	Total Sample Size
BJ119	PV740919	Malaysia: Banjar	1
p8384	PV741038	PNG: North	1
p8234	PV740992	PNG: Lowlands	11
p8276	PV741001		
p8285	PV741004		
p8364	PV741032		
p8385	PV741039		
p8399	PV741043		
p8408	PV741045		
p8414	PV741046		
p8416	PV741047		
p8456	PV741051		
p8471	PV741055		
p8203	PV740987	PNG: Lowlands, Central	41
p8211	PV740988		
p8212	PV740989		
p8218	PV740990		
p8228	PV740991		
p8235	PV740993		
p8242	PV740994		
p8244	PV740995		
p8245	PV740996		
p8266	PV740998		
p8297	PV741006		
p8302	PV741009		
p8309	PV741012		
p8311	PV741013		
p8314	PV741015		
p8315	PV741016		
p8318	PV741017		
p8322	PV741019		
p8324	PV741020		
p8325	PV741021		
p8328	PV741023		
p8330	PV741024		
p8331	PV741025		
p8333	PV741026		
p8334	PV741027		
p8348	PV741030		
p8354	PV741031		
p8365	PV741033		
p8376	PV741034		
p8379	PV741036		
p8383	PV741037		
p8388	PV741040		
p8406	PV741044		
p8424	PV741048		
p8438	PV741049		
p8448	PV741050		
p8462	PV741052		
p8463	PV741053		
p8464	PV741054		
p8479	PV741056		
p8578	PV741061		
p8012	PV740985	PNG: Lowlands, East Sepik	1
p8274	PV741000	PNG: Lowlands, Gulf	9
p8292	PV741005		
p8300	PV741007		
p8303	PV741010		
p8319	PV741018		
p8327	PV741022		
p8377	PV741035		
p8390	PV741041		
p8398	PV741042		

Sample ID	GenBank accession number	Location	Total Sample Size
p7941	PV740984	PNG: Lowlands, Madang	6
p8336	PV741028		
p8498	PV741057		
p8529	PV741058		
p8577	PV741060		
p8592	PV741063		
p8537	PV741059	PNG: Lowlands, Mix	1
p7843	PV740983	PNG: Lowlands, Morobe	3
p8308	PV741011		
p8585	PV741062		
p8284	PV741003	PNG: Lowlands, North	2
p8313	PV741014		
p8252	PV740997	PNG: Milne Bay	2
p8277	PV741002		
p8269	PV740999	Bismarcks: New Britain	1
p8067	PV740986	Bismarcks: New Ireland	1
p8301	PV741008	Manus	2
p8337	PV741029		
6615	PV740830	Solomon Islands	19
6616	PV740831		
6619	PV740832		
6621	PV740833		
6622	PV740834		
6623	PV740835		
6624	PV740836		
6626	PV740837		
6627	PV740838		
6629	PV740839		
6630	PV740840		
6631	PV740841		
6632	PV740842		
6634	PV740843		
6636	PV740844		
6637	PV740845		
6639	PV740846		
6641	PV740847		
6642	PV740848		
GUA105	PV740921	Guam	3
GUA107	PV740922		
Gua74_R1	PV740923		
KB57	PV740926	Kiribati	6
KB80	PV740927		
KB95	PV740929		
KB117	PV740924		
KB120	PV740925		
KB86	PV740928		
NAU37	PV740953	Micronesia: Nauru	18
NAU47	PV740957		
NAU08	PV740946		
NAU10	PV740947		
NAU25	PV740948		
NAU32	PV740949		
NAU34	PV740950		
NAU35	PV740951		
NAU36	PV740952		
NAU38	PV740954		
NAU39	PV740955		
NAU42	PV740956		
NAU48	PV740958		
NAU49	PV740959		
NAU51	PV740960		
NAU52	PV740961		
NAU56	PV740962		
NAU62	PV740963		

Sample ID	GenBank accession number	Location	Total Sample Size
ATN23	PV740854	Vanuatu	3
ATN26	PV740855		
ATN29	PV740856		
ATN4	PV740858	Vanuatu: Ambrym	3
B136	PV740905		
B154	PV740911		
AN106	PV740849	Vanuatu: Aneityum	4
AN155	PV740850		
AN219	PV740851		
AN250	PV740852		
B134	PV740903	Vanuatu: Aoba	5
B135	PV740904		
B141	PV740907		
B83	PV740916		
B88	PV740917		
BT1054	PV740920	Vanuatu: Banks and Torres	1
B133	PV740902	Vanuatu: Efate	12
B145	PV740908		
B149	PV740910		
B91	PV740918		
LS140	PV740930		
LS141	PV740931		
LS142	PV740932		
LS145	PV740933		
LS149	PV740934		
LS151	PV740935		
LS153	PV740936		
LS155	PV740937		
ATR115	PV740860	Vanuatu: Emae	6
ATR69	PV740886		
ATR73	PV740887		
ATR74	PV740888		
ATR76	PV740889		
ATR79	PV740890		
B132	PV740901	Vanuatu: Erromango	1
B148	PV740909	Vanuatu: Futuna	1
MB50	PV740938	Vanuatu: Maewo	4
MB82	PV740939		
MB88	PV740940		
MB91	PV740941		
ATR128	PV740861	Vanuatu: Makura	5
ATR129	PV740862		
ATR136	PV740863		
ATR142	PV740864		
ATR143	PV740865		
ATN21	PV740853	Vanuatu: Malekula	2
B74Malekula	PV740913		
ATN31	PV740857	Vanuatu: Mota Rava	1
B104	PV740898	Vanuatu: Paama	3
B122	PV740899		
B138	PV740906		

Sample ID	GenBank accession number	Location	Total Sample Size
ATN6	PV740859	Vanuatu: Pentecost	13
ATR231	PV740875		
ATR240	PV740876		
ATR242	PV740877		
ATR252	PV740878		
ATR256	PV740879		
ATR259	PV740880		
ATR263	PV740881		
ATR270	PV740882		
ATR271	PV740883		
ATR296	PV740884		
ATR343	PV740885		
B158	PV740912		
ATR158	PV740866	Vanuatu: Santo	26
ATR161	PV740867		
ATR169	PV740868		
ATR186	PV740869		
ATR187	PV740870		
ATR188	PV740871		
ATR200	PV740872		
ATR201	PV740873		
ATR203	PV740874		
B74Santo	PV740914		
B8	PV740915		
N163	PV740942		
N173	PV740943		
N176	PV740944		
N200	PV740945		
NR41	PV740964		
OK11	PV740965		
OK38	PV740966		
PO110	PV740967		
PO60	PV740968		
SR4	PV740969		
T151	PV740970		
T164	PV740971		
T169	PV740972		
T191	PV740973		
T208	PV740974		
B123	PV740900	Vanuatu: Tongariki	1
TAV12	PV740975	Fiji	8
TAV13	PV740976		
TAV16B	PV740977		
TAV17B	PV740978		
TAV24	PV740979		
TAV26	PV740980		
TAV2B	PV740981		
TAV30	PV740982		
Ati10	PV740891	Cook Islands	7
Ati12	PV740892		
Ati13	PV740893		
Ati17	PV740894		
Ati21	PV740896		
Ati2	PV740895		
Ati6	PV740897		

Supplementary Table 4. Genetic estimates performed in this paper. PNG North Coast and New Ireland were the source regions for the founder analysis, so no founder ages are shown for these regions. We also provide an overall age estimate for the Polynesian motif (PM), based on all Near Oceania data excluding New Britain. Age estimates and founder ages (including confidence intervals) were rounded to the nearest 10 years for improved readability. Sites with fewer than 15 samples and ancient DNA were removed from the analyses.

	**Nucleotide Diversity (π)**					**Rho (ρ)**					**Founder Analysis**				
**Islands**	Number of Sequences	Number of Segregating Sites	Number of Haplotypes	Haplotype Diversity	Nucleotide Diversity	Diversity Measure	Standard Deviation	Age (BP)	Lower Bound (Years BP)	Upper Bound (Years BP)	Diversity Measure	Standard Deviation	Age (BP)	Lower Bound (Years BP)	Upper Bound (Years BP)
PM overall (Near Oceania)	806	718	706	0.994	0.00033	2.482	0.456	6500	4130	8900	-	-	-	-	-
ISEA	29	52	26	0.990	0.00036	1.966	0.303	5120	3560	6710	1.655	0.276	**4300**	2880	5740
PNG: North Coast	29	45	29	1.000	0.00034	2.464	0.586	6450	3410	9550	-	-	-	-	-
PNG: Central Lowlands	57	70	45	0.985	0.00028	2.123	0.399	5540	3480	7630	1.368	0.186	**3550**	2600	4510
PNG: Southeast Coast	18	32	18	1.000	0.00034	2.389	0.674	6250	2760	9810	1.333	0.304	**3460**	1900	5030
New Britain	42	32	27	0.974	0.00023	1.643	0.507	4270	1670	6910	1.143	0.325	**2960**	1300	4630
New Ireland	26	54	25	0.997	0.00035	2.769	0.560	7270	4350	10250	-	-	-	-	-
Bougainville	121	92	73	0.983	0.0003	2.603	0.571	6830	3850	9850	1.851	0.327	**4820**	3130	6530
Solomon Islands	555	298	244	1	0.00026	2.268	0.443	5930	3630	8260	1.427	0.145	**3700**	2960	4450
Micronesia	28	35	24	0.989	0.00033	2.250	0.607	5880	2740	9080	1.500	0.449	**3900**	1600	6230
Vanuatu	103	99	73	0.988	0.00025	1.854	0.300	4830	3280	6390	1.330	0.187	**3450**	2490	4410
Fiji	45	44	31	0.974	0.00022	1.933	0.477	5040	2580	7540	1.000	0.192	**2590**	1610	3570
Futuna	42	41	18	0.919	0.00028	2.286	0.490	5980	3430	8560	1.262	0.322	**3270**	1630	4930
Tuvalu	43	37	26	0.958	0.00025	2.256	0.604	5900	2770	9080	0.953	0.227	**2460**	1310	3630
Samoa	45	68	38	0.987	0.00031	2.333	0.369	6100	4190	8040	1.111	0.175	**2880**	1980	3770
Tonga	47	73	43	0.995	0.00031	2.596	0.454	6800	4440	9210	0.979	0.166	**2530**	1680	3380
Niue	21	15	12	0.905	0.00019	1.524	0.551	3960	1140	6830	0.238	0.126	**610**	0	1250
Cook Islands	70	47	38	0.953	0.00031	1.214	0.303	3150	1600	4710	0.586	0.130	**1510**	850	2170
New Zealand	20	21	13	0.911	0.00026	2.650	0.743	6950	3090	10900	0.800	0.255	**2070**	770	3370
Remote Oceania	467	304	271	0.987	0.0003	2.055	0.322	5360	3700	7050	1.244	0.098	**3220**	2720	3730
